# HCX3 Mitigates LPS-Induced Inflammatory Responses in Macrophages by Suppressing the Activation of the NF-κB Signaling Pathway

**DOI:** 10.3390/cimb47100809

**Published:** 2025-10-01

**Authors:** Qianyi Wu, Jiyuan Shi, Luojin Wu, Lingxi Li, Yong Ling, Liming Mao, Jie Zhang

**Affiliations:** 1 Department of Immunology, School of Medicine, Nantong University, 19 Qixiu Road, Nantong 226001, China; 2331310018@stmail.ntu.edu.cn (Q.W.); 2331310021@stmail.ntu.edu.cn (J.S.); 2431310023@stmail.ntu.edu.cn (L.W.); 2331110020@stmail.ntu.edu.cn (L.L.); 2 School of Pharmacy and Jiangsu Province Key Laboratory for Inflammation and Molecular Drug Target, Nantong University, Nantong 226001, China; lyyy111@sina.com; 3 Basic Medical Research Center, School of Medicine, Nantong University, Nantong 226019, China

**Keywords:** HCX3, inflammation, acute lung injury, LPS, NF-κB, macrophages

## Abstract

Acute lung injury (ALI) is a severe pulmonary disorder characterized by the disruption of the alveolar–capillary barrier, leading to impaired oxygenation and pulmonary edema. Current pharmacological interventions primarily involve the use of steroid drugs, oxygen radical scavengers, and bronchodilators. However, the therapeutic efficacy of these interventions remains inconsistent. Canthin-6-ones, a class of tryptophan-derived alkaloids, exhibit anti-inflammatory, antioxidant, and immunomodulatory properties. In this study, we synthesized a novel Canthin-6-one derivative, namely HCX3, and evaluated its potential beneficial effects and underlying mechanisms on ALI. Prior to the experimental study, network pharmacology analysis revealed that HCX3 may exert anti-inflammatory effects in the context of ALI through the regulation of multiple signaling pathways, including the NF-κB pathways. To validate these findings, Lipopolysaccharide (LPS) was employed to stimulate RAW 264.7 macrophages and bone marrow-derived macrophages (BMDMs) to construct cellular models of inflammatory response associated with ALI. Our data demonstrated that exposure to HCX3 significantly inhibited the transcription and the secretion of multiple pro-inflammatory mediators, including IL-1β, IL-6, and TNF-α, in a dose-dependent manner. Additionally, HCX3 reduced LPS-induced phosphorylation levels of p65 and IκB-α in macrophages, indicating an inhibitory effect of the compound on the activation of NF-κB signaling pathway. Collectively, our data suggest that HCX3 exhibits significant anti-inflammatory effects by inhibiting NF-κB-related signaling pathways, providing new insights for ALI treatment.

## 1. Introduction

Acute lung injury (ALI) is an inflammatory condition of the lungs associated with high morbidity and mortality [1]. Despite extensive clinical studies, it still has a mortality rate of 30% to 50% [2]. It is characterized by uncontrolled pulmonary edema, oxidative stress (OS), and infiltration of inflammatory cells [3]. Most diseases are related to OS, which is caused by excess ROS [4]. ALI can be triggered by a variety of etiological factors [5], with the most common being severe infections such as sepsis/septic shock and pneumonia caused by microbial pathogens, including bacteria, viruses, fungi, rickettsia, and parasites [6]. Lipopolysaccharide (LPS), a complex molecule located in the outer membrane of Gram-negative bacteria, serves as a major microbial mediator in infectious diseases and has been widely used in the research of inflammatory diseases [7,8]. Upon binding to TLR4, LPS activates myeloid differentiation factor 88 (MyD88)-dependent signaling pathway, subsequently leading to the activation of nuclear factor kappa B (NF-κB). NF-κB, as the main transcription factor in the inflammatory process, reacts first when cells are stimulated by LPS, resulting in relatively rapid changes in the expression of corresponding target genes [9]. As a persistent disease, ALI produces inflammation that plays an important role in the development and progression of the disease. When inflammation is poorly controlled, pro-inflammatory mediators are overexpressed, leading to pathological lesions [10]. NF-κB regulates the expression of pro-inflammatory cytokines such as tumor necrosis factor α (TNF-α), interleukin 1β (IL-1β), and interleukin 6 (IL-6) [11]. Furthermore, NF-κΒ signaling induces the expression of genes involved in regulating oxidative stress and mucus production, thereby promoting inflammation and increasing pulmonary permeability, eventually contributing to the development of ALI [12].

Due to the incomplete understanding of the pathophysiology and pathogenesis of ALI, there is currently no specific therapeutic treatment available for this condition [13]. Similarly to many refractory diseases, the purpose of clinical treatment is now to delay the deterioration of lung function, improve quality of life, and delay disease progression [14]. Standard clinical management includes mechanical ventilation, fluid management, surfactant replacement therapy, glucocorticoid administration, inhaled vasodilators, and extracorporeal membrane oxygenation [15]. On this basis, patients can delay the progression of the disease with regular exercise [16]. However, studies have demonstrated that commonly prescribed therapeutic agents, such as antibiotics and glucocorticoids, are associated with significant adverse effects and potential dependency, which limits their long-term applicability. Therefore, it is of great significance to investigate the underlying mechanism of ALI and identify novel therapeutic drugs for the treatment of ALI.

**Figure 1:**
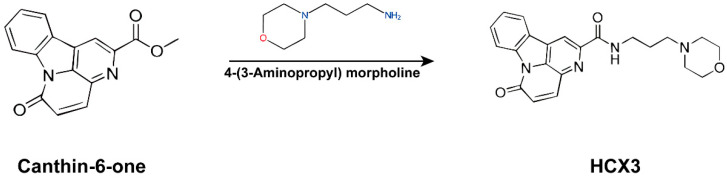
The chemical structure of canthin-6-one, 4-(3-aminopropyl) morpholine and HCX3.

Canthin-6-one is an indole alkaloid widely distributed in various plants, such as Picrasma quassiodes (D.Don) Benn. (PQ) [7], a medicinal herb commonly found throughout most regions of the Chinese mainland and predominantly utilized in treating various diseases, including colitis, hypertension, gastroenteritis, and cancer [17,18,19]. Studies have demonstrated that Canthin-6-one exhibits anti-inflammatory, antioxidant, and hypolipidemic effects [20,21]. However, its potential role in ALI has not been explored. In this study, we reprocessed canthin-6-one to generate a novel derivative, HCX3 (Figure 1). Specifically, an aromatic ring and an amino propyl morpholine group were introduced into the canthin-6-one scaffold. These structural modifications are expected to enhance water solubility and bioavailability, improve target binding affinity, increase the capacity to regulate specific signaling pathways, and promote favorable in vivo distribution and metabolic stability. We investigated the effect of HCX3 on LPS-mediated inflammatory responses in macrophages, which are critically associated with the progression of ALI. Our data suggest that HCX3 treatment significantly inhibits the production of pro-inflammatory cytokines by macrophages and may mitigate the development of ALI by inhibiting the NF-κB signaling pathway. Therefore, HCX3 represents a promising candidate for the development of novel therapeutics for the treatment of ALI.

## 2. Materials and Methods

### 2.1. Materials and Reagents

HCX3 was generated according to the literature [22]. Lipopolysaccharide (LPS, tlrl-b5lps, InvivoGen, Santiago, CA, USA) was purchased from InvivoGen. Dexamethasone (DEX, D829854, Macklin, Wuhan, China) was commonly used by many studies and was used as a positive control drug for lung inflammation [23,24,25].

### 2.2. Cell Culture

Bone-marrow-derived macrophages (BMDMs) were isolated from the femur and tibia of 8-week-old male C57BL/6J mice [26]. The specific method is as follows: the mice were subjected to euthanasia in a CO_2_ cabinet, the tibia and femur were separated under sterile conditions. The surrounding tissue was further isolated and removed. The bony ends of the tibia and femur were cut with eye-cutting scissors, the complete medium was aspirated with a syringe, and the bone marrow cells were washed into a centrifuge tube. The complete medium used here is IMDM medium (12440053, Gibco, Grand Island, NY, USA) with 10% FBS (10099141C, Gibco, Grand Island, NY, USA) and 1% penicillin-streptomycin (HyClone, Logan, UT, USA). Repeat this process until the bones turn white. Red blood cell lysis buffer (C3702, Beyotime, Shanghai, China) is then used in combination with a cell strainer to remove red blood cells, tissue clumps, and other impurities. After centrifugation collection, cells were suspended in IMDM medium (12440053, Gibco, Grand Island, NY, USA) containing 10 S (10099141C, Gibco, Grand Island, NY, USA) supplemented with 10 ng/mL M-CSF (315-02, Peprotech, Rocky Hill, NJ, USA) to promote myeloid cell differentiation into macrophages. Then adjust the cell density to 1 × 10^6^ cells/mL and seed the cells in a dish to wait for 7 days for further use. Mouse RAW 264.7 cells were purchased from Hefei Wanwu Biotechnology Co., Ltd. (Tings-12733, Hefei, China) and cultured in DMEM high-sugar medium containing 10% (FBS-S500, Newzerum, Christchurch, New Zealand) and 1% penicillin-streptomycin (HyClone, Logan, UT, USA). The experiment is divided into 5 groups: control group, model group, HCX3 low dose group, HCX3 high dose group, and positive drug control group. All cells were allocated to different groups using a completely randomized method before inoculation. The above cells were grown in an incubator containing 5% CO_2_ at 37 °C. The animal care and experimental procedures for this study have been approved by the Institutional Animal Care and Use Committee of Nantong University (Approval No. S20250623-006, Approved on 16 February 2022).

### 2.3. Network Pharmacology

The ZINC (https://zinc.docking.org/ (accessed on 6 June 2025)) [27], BATMAN-TCM (http://bionet.ncpsb.org.cn/batman-tcm/index.php (accessed on 6 June 2025)) [28] and SwissTargetPrediction (http://swisstargetprediction.ch/ accessed on 6 June 2025)) [29] databases were searched for targets associated with HCX3. A thorough search of the GeneCards (https://www.genecards.org/ (accessed on 6 June 2025)) [30], DrugBank (https://www.drugbank.com/datasets (accessed on 6 June 2025)) [31], TTD (https://db.idrblab.net/ttd/ (accessed on 6 June 2025)) [32], PharmGkb (https://www.pharmgkb.org/page/overview (accessed on 6 June 2025)) [33] and OMIM (https://www.omim.org/ (accessed on 6 June 2025)) [34] databases was performed to identify targets associated with ALI for Homo sapiens. After removing duplicates and adopting values with a correlation greater than the average, a different target is left. The Venn diagram is drawn using the R 4.3.2 package ‘Venn’. Open the “TSV” file using Cytoscape 3.10.2 to build the PPi network. The exported network information is a TSV file. Obtain network diagram related data through topology analysis. The Weisheng Xin platform was used to perform enrichment analysis of the main nodes of ALI and HCX3, including the Kyoto Encyclopedia of Genes and Genomes (KEGG) and Gene Ontology (GO). Data is visually represented using bar charts or bubble charts.

### 2.4. CCK8 Assay

Cell counting kit (CCK8 kit, abs50003—5 mL, Absinthe, Shanghai, China) was used to assess cell viability with or without LPS stimulation. Cells were counted and adjusted to a concentration of 5000 cells/mL, and then seeded in 96-well plates at a concentration of 100 μL/well. Cells from each treatment group are seeded in three replicate wells. The time required to incubate the cells in a cell culture incubator at 37 °C and 5% CO_2_. Add 10 μL of CCK8 solution to each well. Place the cell culture plate in the incubator for 1–4 h. Finally, measure the absorbance at 450 nm using a plate reader. As control wells, untreated cells, culture medium, and CCK8 solution were also examined.

### 2.5. Western Blot

The cells were washed with PBS at room temperature before lysing in 1% NP40 solution. Protein concentrations in lysed samples were determined and their concentrations were uniformly adjusted. Here, we used the BCA protein concentration detection kit (U10007A, UUBIO, Suzhou, China) to adjust the protein concentration of different samples. The protein samples were then heated at 99 °C for 10 min using a thermostatic heater and loaded into a 4–15% SDS-polyacrylamide gel (P0466M, Beyotime, Shanghai, China). After the gel running, the proteins were transferred to the NC membrane (Absinthe, China) using a semi-dry transfer method (Bio-Rad Laboratories GmbH, München, Germany). The NC membrane was then closed in 5% skimmed milk powder (P0216, Beyotime, Shanghai, China) for 2 h at room temperature. Membranes were incubated in solutions containing primary antibodies recognizing iNOS (#ER1706-89, HUABIO, Hangzhou, China), COX-2 (#PT0297R, Immunoway, Beijing, China), p65 (#8242, Cell Signaling Technology, Boston, MA, USA), p-p65 (#3033, Cell Signaling Technology, Boston, MA, USA), IKB-α (#9242, Cell Signaling Technology, Boston, MA, USA). p-IKB-α (#9246, Cell Signaling Technology, Boston, MA, USA) and β-actin (#ET1702-67, HUABIO, China). After incubating at 4 °C overnight, the NC membranes were incubated in solutions containing horseradish peroxidase (HRP)-conjugated secondary antibodies (Beyotime, Shanghai, China) at room temperature for 1 h. Signal intensity was detected using WesternBright^TM^ Sirius (K-12043-D10, Menlo Park, CA, USA) using the Tanon gel imager. Each Western blot experiment was conducted with at least three independent biological replicates, and the representative blot shown is from one of the replicates, with results expressed as mean ± SEM.

### 2.6. ELISA

The concentration of the cytokine IL-6 was determined using a commercial ELISA kit according to the manufacturer’s protocol (550950, BD Biosciences, San Jose, CA, USA). Absorbance was read at 450 nm with a correction wavelength of 630 nm.

### 2.7. Quantitative Polymerase Chain Reaction (qPCR)

The RNeasy Mini Kit (74104, Qiagen, Germantown, MD, USA) was utilized to extract the total cellular RNA of RAW 264.7 cells and BMDMs. The RevertAid First Strand cDNA Synthesis kit (K1622, Thermofisher, Waltham, MA, USA) was used to create complementary DNA (cDNA) samples. Following the directions on the SYBR Green RT-PCR reaction kit (A25742, Thermofisher, Waltham, MA, USA), cDNA was then amplified. The relative quantitative analysis of 2^−ΔΔCT^ was used to calculate the test findings. Every test was run three times. The sequences of the primers used in this experiment are detailed in Table 1:

**Table 1:** The primers used in qPCR.

Gene	Forward	Reverse
GAPDH	AGGTCGGTGTGAACGGATTTG	TGTAGACCATGTAGTTGAGGTCA
IL-1β	GCAACTGTTCCTGAACTCAACT	ATCTTTTGGGGTCCGTCAACT
IL-6	TAGTCCTTCCTACCCCAATTTCC	TTGGTCCTTAGCCACTCCTTC
TNF-α	CCCTCACACTCAGATCATCTTCT	GCTACGACGTGGGCTACAG

### 2.8. Molecular Docking

The three-dimensional (3D) structures of TLR4, MyD88, IκBα, and p65 were retrieved from the Protein Data Bank (PDB, https://www.rcsb.org/ (accessed on 17 September 2025) [35]. Prior to molecular docking, all retrieved protein structures were preprocessed using AutoDock Tools 1.5.7 to ensure optimal conformational states for docking: specifically, solvent water molecules and redundant ligands were removed, hydrogen atoms were added to the protein backbone and side chains, and energy minimization was performed (using the Amber ff14SB force field) to eliminate steric clashes and stabilize the protein structure. The two-dimensional (2D) structures of the relevant molecules were downloaded as Structure Data Format (SDF) files from the PubChem (https://pubchem.ncbi.nlm.nih.gov/, accessed on 17 September 2025) [36]. These 2D structures were then converted to energy-minimized 3D conformations using ChemBio3D 14.0 (with the MM2 force field for geometry optimization). Molecular docking simulations were carried out using AutoDock Vina, and the resulting docking complexes (protein-ligand interactions) were visualized and analyzed using PyMOL 3.1.6.1.

### 2.9. Statistical Analysis

All statistical analyses in this study were conducted using GraphPad Prism 9.5 software (La Jolla, CA, USA). The differences between multiple groups were analyzed using ANOVA. A significance threshold of *p* < 0.05 was applied to determine statistical significance.

## 3. Results

### 3.1. Network Pharmacological Analysis Reveals a Potential Role of HCX3 in Modulating Inflammatory Responses

**Figure 2:**
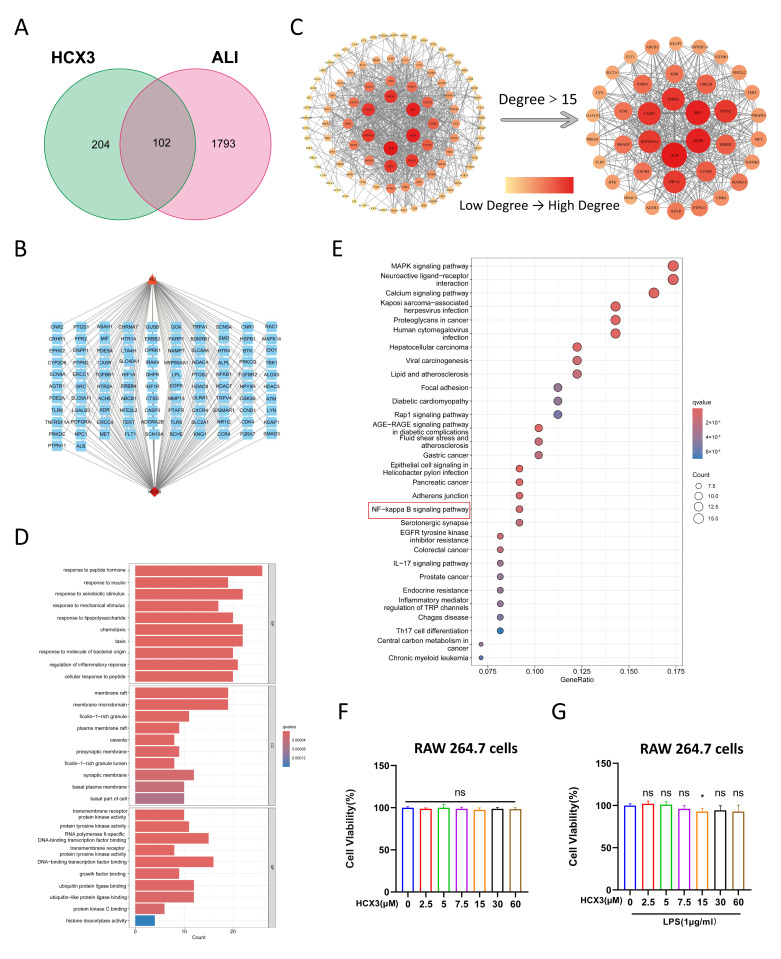
Pharmacological analysis reveals that HCX3 exhibits potential anti-ALI effects. (**A**) HCX3 target genes were retrieved from multiple databases and intersected with ALI-related genes. (**B**) The HCX3-ALI-target networks was constructed and visualized using Cytoscape 3.10.2 software. (**C**) A PPI network was established to illustrate the target interactions and identify hub proteins. (**D**) GO enrichment analysis identified significantly enriched BPs, CCs, and MFs associated with HCX3 targets. (**E**) KEGG pathway analysis revealed enriched signaling pathways associated with HCX3 target genes. (**F**) RAW 264.7 cells were exposed to varying concentrations of HCX3, and then cell viability was assessed using the CCK8 assay after 24 h. (**G**) RAW 264.7 cells were exposed to varying concentrations of HCX3, followed by LPS stimulation, and cell viability was evaluated using CCK8 assay after 24 h. Data in (**F**,**G**) are expressed as mean ± SEM (*n* = 3) (* = *p* < 0.05, ns = not significant compared to the control group).

Prior to experimental validation of HCX3’s effects, we performed a network pharmacology analysis to evaluate its potential involvement in inflammatory responses associated with ALI. A total of 306 HCX3-targeted genes were retrieved from ZINC, BATMAN-TCM, and SwissTargetPrediction databases. Concurrently, a systematic search of the GeneCards, DrugBank, TTD, PharmGkb, and OMIM databases using the keyword “acute lung injury” identified 1895 ALI-related genes, after excluding duplicates. Using the R 4.3.2 package “Venn”, we identified 102 overlapping genes between HCX3 target genes and ALI-related genes (Figure 2A). Subsequently, a HCX3-ALI-target interaction network was constructed using Cytoscape 3.10.2 software (Figure 2B). Additionally, a PPI network was established to visualize the interactions among these targets, with those having a node degree greater than 15 highlighted separately (Figure 2C). Gene Ontology (GO) enrichment analysis revealed 2015 statistically significant GO terms, comprising 1816 BPs, 61 CCs, and 139 MFs. The top 10 enriched GO terms for each category are presented in Figure 2D. To further understand the potential mechanisms underlying HCX3-mediated anti-inflammatory effects, KEGG pathway enrichment analysis was performed, revealing significant enrichment in multiple signaling pathways (Figure 2E). These findings suggest that HCX3 may exert anti-inflammatory effects in the context of ALI through the regulation of key signaling pathways, including the MAPK and NF-κB signaling pathways.

### 3.2. The Effect of HCX3 on Cell Viability of Macrophages

Based on the results of the aforementioned network pharmacology analysis, we further evaluated the potential effects of HCX3 on macrophage-mediated inflammatory responses, which are critically involved in the progression of ALI. Prior to assessing its anti-inflammatory potential, we first evaluated the potential cytotoxicity of HCX3 in vitro. To achieve this, RAW 264.7 cells, a murine macrophage cell line, were treated with varying concentrations of HCX3, and cell viability was measured after 24 h using the CKK8 assay. The results indicated that HCX3 did not exhibit cytotoxic effects at concentrations up to 60 μM over 24 h (Figure 2F). Therefore, HCX3 concentrations below 60 μM were selected for subsequent experimental procedures. To establish an inflammatory cell model, RAW 264.7 cells were stimulated with LPS, a widely accepted in vitro model for studying the inflammatory response associated with ALI in existing literature [5]. The dosage and duration of LPS stimulation were determined based on previous studies investigating macrophage-derived pro-inflammatory mediators [37,37]. The results of the CCK8 assay further confirmed that LPS stimulation did not significantly affect macrophage viability within 24 h time frame when HCX3 concentrations remained below 60 μM (Figure 2G).

### 3.3. HCX3 Inhibits the Production of Pro-Inflammatory Mediators by Macrophages

**Figure 3:**
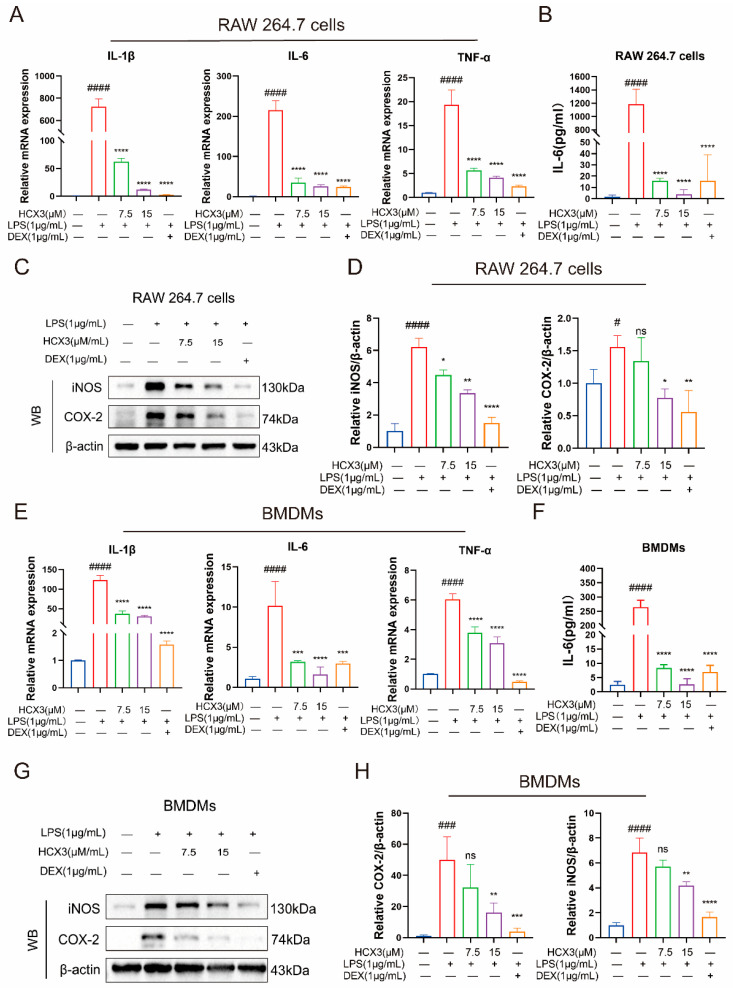
HCX3 inhibits the production of pro-inflammatory mediators. (**A**) RAW 264.7 cells were exposed to indicated concentrations of HCX3 or 1 μg/mL DEX for 1 h, and then stimulated with LPS. Total RNA was extracted from the cells after 24 h, and the transcription levels of IL-1β, IL-6, and TNF-α were subsequently detected by RT-PCR. (**B**) RAW 264.7 cells were treated as indicated in (**A**), collect the culture supernatants after 6 h, and IL-6 levels were detected by ELISA. (**C**,**D**) Following the treatment protocol outlined in (**A**) and the stimulation time is 6 h, cells were lysed and the expression of iNOS and COX-2 were assessed by Western blot, with β-actin used as the internal control. (**E**,**G**,**H**) The results obtained in RAW 264.7 cells were validated using mouse BMDMs. Data in (**A**,**B**,**D**,**E**,**F**,**H**) are presented as mean ± SEM (*n* = 3) (ns = not significant, * vs. LPS group, # vs. Control group, * *p* < 0.05; ** *p* < 0.01; *** *p* < 0.001; **** *p* < 0.0001; # *p* < 0.05, ### *p* < 0.001; #### *p* < 0.0001).

Previous studies have demonstrated that LPS activates downstream signaling pathways by binding to TLR4, ultimately leading to the activation of NF-κB and subsequent initiation of immune and inflammatory responses [38]. Our network pharmacology analysis also suggests that HCX3 may exert its anti-inflammatory effects through modulation of the NF-κB signaling pathway. Accordingly, in our preliminary experiments, we explored the role of HCX3 in regulating the expression of pro-inflammatory cytokines, as their production is predominantly dependent on NF-κB activation. To achieve this, RAW 264.7 cells were pretreated with varying concentrations of HCX3 followed by LPS stimulation. DEX was selected as a positive control drug. Our results revealed that HCX3 significantly inhibited the transcriptional levels of LPS-induced IL-1β, IL-6, and TNF-α in a dose-dependent manner (Figure 3A). Additionally, we collected cell culture supernatants to quantify IL-6 secretion levels, which further supported our findings (Figure 3B). Western blot analysis demonstrated that LPS stimulation increased the expression levels of iNOS and COX-2 (Figure 3C,D), which were attenuated by HCX3 pretreatment. To validate these observations, we utilized primary bone marrow-derived macrophages (BMDMs) in subsequent experiments. Notably, HCX3 also inhibited the production of pro-inflammatory mediators in LPS-stimulated BMDMs (Figure 3E–H). Collectively, these findings indicate that HCX3 has an inhibitory effect on LPS-induced immune responses in macrophages.

### 3.4. HCX3 Inhibits the NF-κB Signaling Pathway

**Figure 4:**
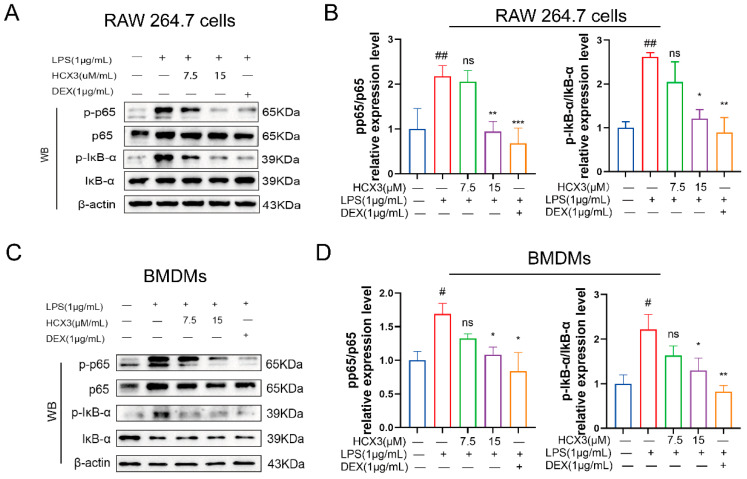
HCX3 inhibits the activation of the NF-κB signaling pathway. (**A**) RAW 264.7 cells were pretreated with the indicated concentrations of HCX3 or 1 μg/mL DEX for 1 h, followed by LPS stimulation for 3 h. Subsequently, the cells were lysed and the phosphorylation levels of p65 and IκB-α, as well as total p65 and IκB-α, were detected using Western blotting. β-actin was used as an internal control. (**B**) Quantitative analysis of phosphorylated p65 and IκB-α relative to their non-phosphorylated forms from (**A**). The same stimulation method was applied to BMDM cells, and the same proteins was detected using Western blotting (**C**). The quantitative data were shown in (**D**). The representative Western blot images in (**A**,**C**) are from three independent experiments. Data in (**B**,**D**) are presented as mean ± SEM (*n* = 3) (ns = not significant, * vs. LPS group, # vs. Control group, * *p* < 0.05; ** *p* < 0.01; *** *p* < 0.001; # *p* < 0.05, ## *p* < 0.01.

To further elucidate the mechanism by which HCX3 inhibits the production of pro-inflammatory cytokines, we conducted a series of experiments. Here, RAW 264.7 cells were pre-treated with varying concentrations of HCX3 or 1 μg/mL DEX for 1 h. Subsequently, we stimulated the cells with LPS and harvested the cells after 3 h. Cell lysates were then subjected to Western blot assays to detect the activation of various inflammation-related signaling pathways. Phosphorylation of p65 is a key regulatory step in the NF-κB signaling pathway [39]. As shown in Figure 4A, LPS stimulation increased p65 phosphorylation levels, while exposure to HCX3 significantly reduced this effect in RAW 264.7 cells (Figure 4A,B). Previous studies have shown that upon cellular stimulation, IκB-α undergoes degradation, allowing NF-κB to translocate into the nucleus and initiate transcriptional activation [40]. Accordingly, we examined the expression levels of IκB-α and its phosphorylated form. The results showed that HCX3 significantly inhibited the phosphorylation of IκB-α caused by LPS. In a concurrent study, we extended our investigation to BMDMs and witnessed comparable inhibitory effects of HCX3 on the NF-κB signaling pathway (Figure 4C,D). Collectively, these data suggest that the inhibitory effect of HCX3 on pro-inflammatory mediators is, at least in part, mediated by the inhibition of the NF-κB signaling pathway.

### 3.5. HCX3 Is Molecularly Docked to Key Proteins of the NF-κB Signaling Pathway

**Figure 5:**
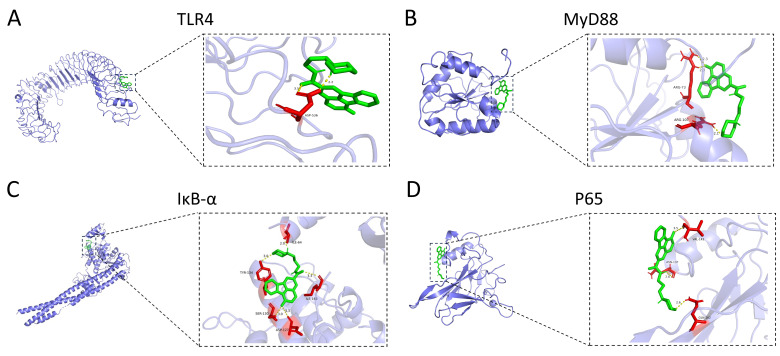
HCX3 is molecularly docked to key proteins of the NF-κB signaling pathway. (**A**–**D**) Results of molecular docking simulations between HCX3 and key proteins of the NF-κB signaling pathway, including TLR4 (**A**), MyD88 (**B**), IκB-α (**C**), and p65 (**D**). In the figure, yellow represents hydrogen bonds, red indicates the relevant amino acid residues of the protein that form hydrogen bonds with molecules, and the numerical values denote the bond lengths. Visualization of the protein-compound complexes (**A**–**D**) illustrates the interaction patterns of HCX3 with the active sites of these key proteins.

**Figure 6:**
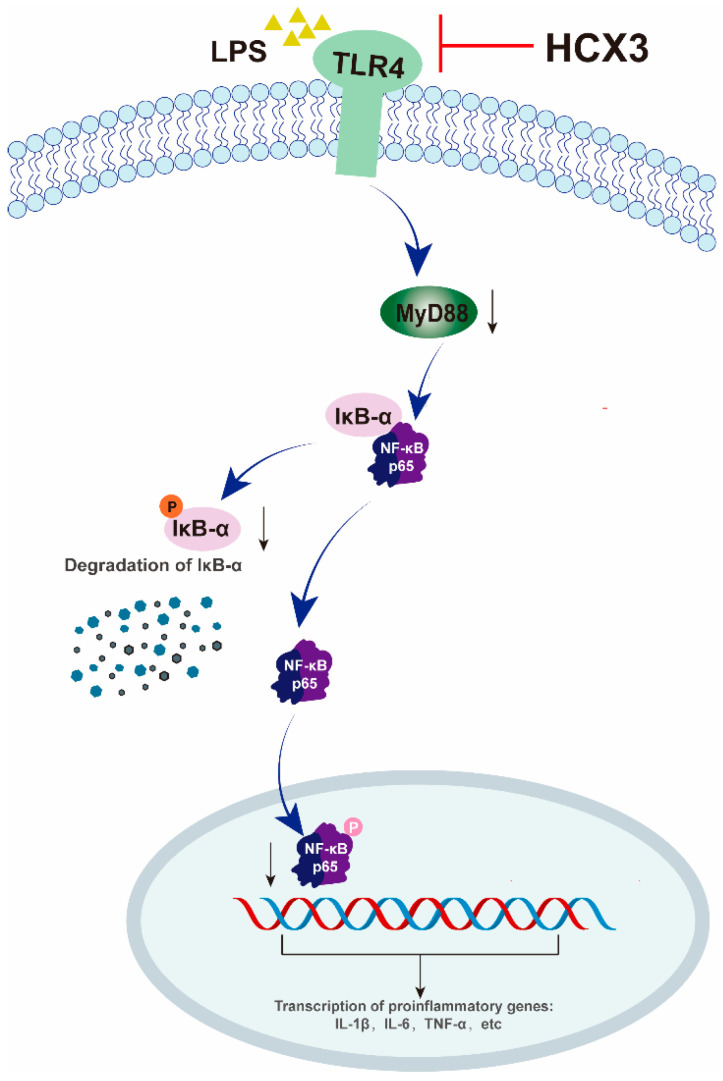
A hypothetical model of the anti-inflammatory mechanism of HCX3. In this model, HCX3 acts on the TLR4/MyD88/NF-κB signaling pathway: the compound inhibits the activation of TLR4 (the primary receptor for LPS), thereby reducing the recruitment of MyD88 to TLR4. This downstream effect further inhibits the activation of signaling proteins such as IκBα and NF-κB p65, as well as the production of inflammatory cytokines. The downward arrow (↓) associated with ‘degradation of IκB-α’ indicates the process by which phosphorylated IκB-α is degraded. The same symbol (↓) in relation to 'MyD88' signifies inhibition of downstream signaling pathways.

To further explore whether HCX3 inhibits the activation of the TLR4-mediated NF-κB signaling pathway by direct binding, molecular docking analyses were performed between HCX3 and TLR4, as well as other key proteins in the NF-κB signaling pathway. In general, a binding energy threshold below −5 kcal/mol is indicative of strong binding affinity [11]. We then visualized four protein-compound complexes (Figure 5A–D). The results revealed that the binding energies of HCX3 with TLR4, MyD88, IκB-α and P65 were −6.7 ± 1 kcal/mol, −6.4 ± 0.5 kcal/mol, −6.75 ± 0.85 kcal/mol, and −7.05 ± 0.55 kcal/mol, respectively. In an attempt to evaluate the accuracy of these results, we performed molecular docking analyses of the four proteins with their respective reference ligands. The results demonstrated that HCX3 exhibits comparable or greater binding affinity toward the four proteins relative to the reference ligands, providing further support for the reliability of the study (Supplementary Figure S1 and Table S1). Collectively, these findings suggest that HCX3 exerts anti-inflammatory properties by binding with key proteins in the TLR4/MyD88/NF-κB signaling pathway and thus blocking its activity (Figure 6).

## 4. Discussion

ALI is frequently associated with high morbidity and mortality and is characterized by severe lung tissue damage, making it a significant global health concern [41]. The immune response plays a central role in understanding the pathophysiological mechanisms of ALI. Upon lung injury, the immune system is activated to eliminate pathogens and protect lung tissue from further damage [42]. However, long-term inflammatory responses can exacerbate lung injury [43]. Clinically, immune dysregulation in patients with ALI amplifies the inflammatory response, resulting in disruption of the pulmonary epithelial barrier [20]. In this study, our findings indicate that HCX3 treatment significantly reduced the levels of pro-inflammatory cytokines in LPS-stimulated RAW 264.7 macrophages and BMDM cells (Figure 3A,E). Additionally, HCX3 downregulated iNOS expression in LPS-induced macrophages (Figure 3C,G). These results demonstrate that HCX3 exerts inhibitory effects on immune responses of macrophages and may protects against LPS-induced ALI.

The innate immune system is the first line of defense of the body, consisting of a physical barrier and cellular and humoral immunity that provides rapid protection against pathogens without generating immune memory [44]. TLRs represent an important class of pattern recognition receptors that play a key role in the innate immune response [45,46]. They are capable of recognizing a wide range of pathogen-associated molecular patterns, which has significant implications for the activation and regulation of innate immunity. As a primary defense mechanism against invading pathogens, TLRs are fundamental in mediating inflammatory responses and modulating immune cell function [47]. Notably, LPS, a component of the outer membrane of Gram-negative bacteria, binds to TLR4 on the surface of macrophages and initiates MyD88-dependent signaling pathways. This pathway activates NF-κB, which induces the transcription of pro-inflammatory cytokines and contributes to inflammatory response and lung tissue injury. These findings align with our results of network pharmacological analysis, which demonstrated significant enrichment of ALI-related genes in the NF-κB signaling pathway (Figure 2E). NF-κB is a crucial transcription factor responsible for the expression of various pro-inflammatory mediators, including IL-1β, TNF-α, and IL-6 [48]. Its downstream target genes are associated with multiple pathological conditions, such as ALI [49]. It is well established that LPS induces nuclear translocation of the NF-κB p65 subunit through IκB degradation [50]. In the present study, we observed a significant reduction in the phosphorylation levels of IκB-α (p-IκB-α/IκB-α ratio) following treatment with different doses of HCX3. Furthermore, phosphorylation of NF-κB and its subsequent nuclear translocation were also reduced, as confirmed by in vitro experiments (Figure 4A,C). Collectively, these findings confirm that HCX3 exerts an anti-inflammatory effects by modulating the NF-κB signaling pathway. However, further investigation is required to identify the specific target protein through which HCX3 exerts its effects.

Although various therapeutic strategies are currently employed in the clinical management of ALI, the overall treatment efficacy remains suboptimal, often accompanied by a range of adverse effects [51]. For example, long-term administration of glucocorticoids may lead to immunosuppression and an increased risk of infection. Glucocorticoids inhibit the body’s immune and inflammatory response, including impairing the phagocytic activity of neutrophils and macrophages and inhibiting the release of cytokines, thereby further compromising the already weakened immune defenses in ALI patients [52]. In this study, we preliminarily verified that HCX3 does not exhibit cytotoxic effects on RAW 264.7 macrophages (Figure 2F,G). In ongoing and future studies, we will further investigate the cytotoxicity of HCX3 in additional cell types relevant to the pathogenesis of acute lung injury and systematically evaluate the therapeutic efficacy and long-term safety profile of multiple HCX3 dosing regimens in animal models of ALI.

This study provides the first evidence that HCX3 exerts anti-inflammatory effects through immunomodulatory mechanisms, with its underlying pathway preliminarily identified as the modulation of the NF-κB signaling pathway. Nevertheless, several limitations should be acknowledged: the current investigation primarily focuses on HCX3’s immunomodulatory mechanisms, without evaluating its specific impact on epithelial barrier integrity. The results of network pharmacology analysis indicate that, in addition to the NF-κB pathway, HCX3 may also act on multiple signaling pathways including MAPK and Nrf2 (Figure 2E); however, this paper did not experimentally validate these pathways. A systematic exploration of the various signaling pathways influenced by HCX3 will help to more comprehensively clarify its mechanism of alleviating disease symptoms, thus the potential roles of these pathways warrant further investigation in subsequent research. Moreover, given the limited range of cell types evaluated and the relatively short development timeline of the experimental models employed in this study, future research should carefully consider the accurate assessment of HCX3’s effects on other cell types involved in the progression of ALI. Additionally, the potential anti-ALI therapeutic efficacy of HCX3 warrants extensive evaluation in animal models to establish robust preclinical evidence supporting its development as a candidate therapeutic agent for ALI. Future research should verify the in vivo effects in an LPS-induced acute lung injury mouse model, including the evaluation of indicators such as lung tissue pathological changes, levels of inflammatory factors, and survival rates. We also need to conduct experiments like qPCR, Elisa, and Western Blot to further validate the mechanism of action of HCX3 in mice. Additionally, a systematic study on the pharmacokinetic characteristics of HCX3, the optimal route of administration and dose–response relationship, and its effects on other relevant cell types such as pulmonary epithelial cells and endothelial cells is necessary. These data will aid in the comprehensive assessment of HCX3’s potential as a candidate drug for the treatment of acute lung injury.

## 5. Conclusions

In summary, we have successfully synthesized HCX3, a derivative of Canthin-6-one, which was identified for the first time as an inhibitory compound of macrophage inflammatory responses. Data obtained from this study demonstrated that HCX3 significantly reduced the production of key inflammatory mediators, such as IL-1β, IL-6, and TNF-α, in LPS-induced ALI cell models. Additionally, HCX3 was found to inhibit the phosphorylation and activation of NF-κB in mouse macrophages. Collectively, these findings indicate that HCX3 holds promise as a potential therapeutic candidate for the treatment of ALI.

## Data Availability

The original contributions presented in this study are included in the article/Supplementary Materials. Further inquiries can be directed to the corresponding authors.

## Supplementary Materials

The following supporting information can be downloaded at: https://www.mdpi.com/article/10.3390/cimb47100809/s1.

## References

[1] Cai J., Xu G., Lin Y., Zhou B., Luo Z., Yu S., Lu J. (2022). Inhibition of TRPV4 attenuates ferroptosis against LPS-induced ALI via Ca^2+^ pathway. Turk. J. Biol..

[2] Matthay M.A., Zemans R.L. (2011). The acute respiratory distress syndrome: Pathogenesis and treatment. Annu. Rev. Pathol..

[3] Derwall M., Martin L., Rossaint R. (2018). The acute respiratory distress syndrome: Pathophysiology, current clinical practice, and emerging therapies. Expert Rev. Respir. Med..

[4] Wang C., Wang W., Dong J., Li X., Ye T., Zeng F., Jiang M., Shi J., Wang X., Zhang L. (2024). Isatin improves oligoasthenospermia caused by busulfan by regulating GSH/GPX4 axis to inhibit ferroptosis. Front. Pharmacol..

[5] Zhou M., Meng L., He Q., Ren C., Li C. (2024). Valsartan attenuates LPS-induced ALI by modulating NF-κB and MAPK pathways. Front. Pharmacol..

[6] Zhang C., Wang X., Wang C., He C., Ma Q., Li J., Wang W., Xu Y.-T., Wang T. (2021). Qingwenzhike Prescription Alleviates Acute Lung Injury Induced by LPS via Inhibiting TLR4/NF-kB Pathway and NLRP3 Inflammasome Activation. Front. Pharmacol..

[7] Lee J.-W., Park J.-W., Shin N.-R., Park S.-Y., Kwon O.-K., Park H.A., Lim Y., Ryu H.W., Yuk H.J., Kim J.H. (2016). *Picrasma quassiodes* (D. Don) Benn. attenuates lipopolysaccharide (LPS)-induced acute lung injury. Int. J. Mol. Med..

[8] Ratajczak M.Z., Kucia M. (2020). SARS-CoV-2 infection and overactivation of Nlrp3 inflammasome as a trigger of cytokine “storm” and risk factor for damage of hematopoietic stem cells. Leukemia.

[9] Volk A., Li J., Xin J., You D., Zhang J., Liu X., Xiao Y., Breslin P., Li Z., Wei W. (2014). Co-inhibition of NF-κB and JNK is synergistic in TNF-expressing human AML. J. Exp. Med..

[10] Ren J., Li L., Wang Y., Zhai J., Chen G., Hu K. (2019). Gambogic acid induces heme oxygenase-1 through Nrf2 signaling pathway and inhibits NF-κB and MAPK activation to reduce inflammation in LPS-activated RAW264.7 cells. Biomed. Pharmacother..

[11] Gan A., Chen H., Lin F., Wang R., Wu B., Yan T., Jia Y. (2025). Sanzi Yangqin Decoction improved acute lung injury by regulating the TLR2-mediated NF-κB/NLRP3 signaling pathway and inhibiting the activation of NLRP3 inflammasome. Phytomedicine Int. J. Phytother. Phytopharm..

[12] Gouda M.M., Shaikh S.B., Bhandary Y.P. (2018). Inflammatory and Fibrinolytic System in Acute Respiratory Distress Syndrome. Lung.

[13] Su R., Zhang Y., Zhang J., Wang H., Luo Y., Chan H.F., Tao Y., Chen Z., Li M. (2021). Nanomedicine to advance the treatment of bacteria-induced acute lung injury. J. Mater. Chem. B.

[14] Zhang Y., Gu L., Xia Q., Tian L., Qi J., Cao M. (2020). Radix Astragali and Radix Angelicae Sinensis in the Treatment of Idiopathic Pulmonary Fibrosis: A Systematic Review and Meta-analysis. Front. Pharmacol..

[15] Hecker M., Walmrath H.-D., Seeger W., Mayer K. (2008). Clinical Aspects of Acute Lung Insufficiency (ALI/TRALI). Transfus. Med. Hemotherapy.

[16] Lu T., Denehy L., Cao Y., Cong Q., Wu E., Granger C.L., Ni J., Edbrooke L. (2020). A 12-Week Multi-Modal Exercise Program: Feasibility of Combined Exercise and Simplified 8-Style Tai Chi Following Lung Cancer Surgery. Integr. Cancer Ther..

[17] Zhao W., Yu J., Su Q., Liang J., Zhao L., Zhang Y., Sun W. (2013). Antihypertensive effects of extract from *Picrasma quassiodes* (D. Don) Benn. in spontaneously hypertensive rats. J. Ethnopharmacol..

[18] Zhao W., Sun C., He J., Chen L., Zhang Y., Sun W. (2013). The possible mechanisms of *Picrasma quassiodes* (D. Don) Benn. in the treatment of colitis induced by 2,4,6-trinitrobenzene sulfonic acid in mice. J. Ethnopharmacol..

[19] Zhang Q., Shu X., Jing F., Wang X., Lin C., Luo A. (2014). Preparative separation of alkaloids from *Picrasma quassioides* (D. Don) Benn. by conventional and pH-zone-refining countercurrent chromatography. Mol. Basel Switz..

[20] Wang Y., Zhao N., Jian Y., Liu Y., Zhao L., He L., Liu Q., Li M. (2022). The pro-inflammatory effect of Staphylokinase contributes to community-associated *Staphylococcus aureus* pneumonia. Commun. Biol..

[21] Zhang Z., Wang A., Wang Y., Sun W., Zhou X., Xu Q., Mao L., Zhang J. (2023). Canthin-6-Ones: Potential Drugs for Chronic Inflammatory Diseases by Targeting Multiple Inflammatory Mediators. Mol. Basel Switz..

[22] Ding J., Sun T., Wu H., Zheng H., Wang S., Wang D., Shan W., Ling Y., Zhang Y. (2023). Novel Canthin-6-one Derivatives: Design, Synthesis, and Their Antiproliferative Activities via Inducing Apoptosis, Deoxyribonucleic Acid Damage, and Ferroptosis. ACS Omega.

[23] Kim H.I., Han Y., Kim M.-H., Boo M., Cho K.-J., Kim H.-L., Lee I.-S., Jung J.H., Kim W., Um J.-Y. (2024). The multi-herbal decoction SH003 alleviates LPS-induced acute lung injury by targeting inflammasome and extracellular traps in neutrophils. Phytomed. Int. J. Phytother. Phytopharm..

[24] Al-Harbi N.O., Imam F., Al-Harbi M.M., Ansari M.A., Zoheir K.M.A., Korashy H.M., Sayed-Ahmed M.M., Attia S.M., Shabanah O.A., Ahmad S.F. (2016). Dexamethasone Attenuates LPS-induced Acute Lung Injury through Inhibition of NF-κB, COX-2, and Pro-inflammatory Mediators. Immunol. Invest..

[25] Liu C., Yin Z., Feng T., Zhang M., Zhou Z., Zhou Y. (2021). An integrated network pharmacology and RNA-Seq approach for exploring the preventive effect of *Lonicerae japonicae* flos on LPS-induced acute lung injury. J. Ethnopharmacol..

[26] Mao L., Dhar A., Meng G., Fuss I., Montgomery-Recht K., Yang Z., Xu Q., Kitani A., Strober W. (2022). Blau syndrome NOD2 mutations result in loss of NOD2 cross-regulatory function. Front. Immunol..

[27] Tingle B.I., Tang K.G., Castanon M., Gutierrez J.J., Khurelbaatar M., Dandarchuluun C., Moroz Y.S., Irwin J.J. (2023). ZINC-22—A Free Multi-Billion-Scale Database of Tangible Compounds for Ligand Discovery. J. Chem. Inf. Model..

[28] Kong X., Liu C., Zhang Z., Cheng M., Mei Z., Li X., Liu P., Diao L., Ma Y., Jiang P. (2024). BATMAN-TCM 2.0: An enhanced integrative database for known and predicted interactions between traditional Chinese medicine ingredients and target proteins. Nucleic Acids Res..

[29] Daina A., Michielin O., Zoete V. (2019). SwissTargetPrediction: Updated data and new features for efficient prediction of protein targets of small molecules. Nucleic Acids Res..

[30] Safran M., Dalah I., Alexander J., Rosen N., Iny Stein T., Shmoish M., Nativ N., Bahir I., Doniger T., Krug H. (2010). GeneCards Version 3: The human gene integrator. Database J. Biol. Databases Curation.

[31] Knox C., Wilson M., Klinger C.M., Franklin M., Oler E., Wilson A., Pon A., Cox J., Chin N.E.L., Strawbridge S.A. (2024). DrugBank 6.0: The DrugBank Knowledgebase for 2024. Nucleic Acids Res..

[32] Zhou Y., Zhang Y., Zhao D., Yu X., Shen X., Zhou Y., Wang S., Qiu Y., Chen Y., Zhu F. (2024). TTD: Therapeutic Target Database describing target druggability information. Nucleic Acids Res..

[33] Barbarino J.M., Whirl-Carrillo M., Altman R.B., Klein T.E. (2018). PharmGKB: A worldwide resource for pharmacogenomic information. Wiley Interdiscip. Rev. Syst. Biol. Med..

[34] Amberger J.S., Bocchini C.A., Schiettecatte F., Scott A.F., Hamosh A. (2015). OMIM.org: Online Mendelian Inheritance in Man (OMIM®), an online catalog of human genes and genetic disorders. Nucleic Acids Res..

[35] Burley S.K., Berman H.M., Kleywegt G.J., Markley J.L., Nakamura H., Velankar S. (2017). Protein Data Bank (PDB): The Single Global Macromolecular Structure Archive. Methods Mol. Biol. Clifton N.J..

[36] Wang Y., Bryant S.H., Cheng T., Wang J., Gindulyte A., Shoemaker B.A., Thiessen P.A., He S., Zhang J. (2017). PubChem BioAssay: 2017 update. Nucleic Acids Res..

[37] Liu Z., Wei J., Sun H., Xu L. (2024). Plumbagin ameliorates LPS-induced acute lung injury by regulating PI3K/AKT/mTOR and Keap1-Nrf2/HO-1 signalling pathways. J. Cell. Mol. Med..

[38] Tang J., Xu L., Zeng Y., Gong F. (2021). Effect of gut microbiota on LPS-induced acute lung injury by regulating the TLR4/NF-kB signaling pathway. Int. Immunopharmacol..

[39] Park M.Y., Ha S.E., Kim H.H., Bhosale P.B., Abusaliya A., Jeong S.H., Park J.-S., Heo J.D., Kim G.S. (2022). Scutellarein Inhibits LPS-Induced Inflammation through NF-κB/MAPKs Signaling Pathway in RAW264.7 Cells. Molecules.

[40] Chen J., Shi X., Deng Y., Dang J., Liu Y., Zhao J., Liang R., Zeng D., Wu W., Xiong Y. (2024). miRNA-148a-containing GMSC-derived EVs modulate Treg/Th17 balance via IKKB/NF-κB pathway and treat a rheumatoid arthritis model. JCI Insight.

[41] Zheng J., Li Y., Kong X., Guo J. (2024). Exploring immune-related pathogenesis in lung injury: Providing new insights into ALI/ARDS. Biomed. Pharmacother. Biomed. Pharmacother..

[42] Yuan Y., Fan G., Liu Y., Liu L., Zhang T., Liu P., Tu Q., Zhang X., Luo S., Yao L. (2022). The transcription factor KLF14 regulates macrophage glycolysis and immune function by inhibiting HK2 in sepsis. Cell. Mol. Immunol..

[43] Han X., Zhao Z.-A., Yan S., Lei W., Wu H., Lu X.-A., Chen Y., Li J., Wang Y., Yu M. (2020). CXADR-like membrane protein protects against heart injury by preventing excessive pyroptosis after myocardial infarction. J. Cell. Mol. Med..

[44] He-Yang J., Zhang W., Liu J., Xue P., Zhou X. (2020). Human breast milk oligosaccharides attenuate necrotizing enterocolitis in rats by suppressing mast cell accumulation, DPPI activity and TLR4 expression in ileum tissue, and regulating mitochondrial damage of Caco-2 cells. Int. Immunopharmacol..

[45] Fitzgerald K.A., Kagan J.C. (2020). Toll-like Receptors and the control of immunity. Cell.

[46] Kawai T., Ikegawa M., Ori D., Akira S. (2024). Decoding Toll-like receptors: Recent insights and perspectives in innate immunity. Immunity.

[47] Jabłońska A., Jabłonowska E., Studzińska M., Kamerys J., Paradowska E. (2021). The TLR9 2848C/T Polymorphism Is Associated with the CMV DNAemia among HIV/CMV Co-Infected Patients. Cells.

[48] Jing W., Chunhua M., Shumin W. (2015). Effects of acteoside on lipopolysaccharide-induced inflammation in acute lung injury via regulation of NF-κB pathway in vivo and in vitro. Toxicol. Appl. Pharmacol..

[49] Shin N.-R., Shin I.-S., Song H.-H., Hong J.-M., Kwon O.-K., Jeon C.-M., Kim J.-H., Lee S.-W., Lee J.-K., Jin H. (2015). Callicarpa japonica Thunb. reduces inflammatory responses: A mouse model of lipopolysaccharide-induced acute lung injury. Int. Immunopharmacol..

[50] Yeh C.-H., Yang J.-J., Yang M.-L., Li Y.-C., Kuan Y.-H. (2014). Rutin decreases lipopolysaccharide-induced acute lung injury via inhibition of oxidative stress and the MAPK-NF-κB pathway. Free Radic. Biol. Med..

[51] Yi L., Chang M., Zhao Q., Zhou Z., Huang X., Guo F., Huan J. (2020). Genistein-3’-sodium sulphonate protects against lipopolysaccharide-induced lung vascular endothelial cell apoptosis and acute lung injury via BCL-2 signalling. J. Cell. Mol. Med..

[52] Wigenstam E., Elfsmark L., Koch B., Bucht A., Jonasson S. (2016). Acute respiratory changes and pulmonary inflammation involving a pathway of TGF-β1 induction in a rat model of chlorine-induced lung injury. Toxicol. Appl. Pharmacol..

