# Supplementary material for: HCX3 Mitigates LPS-Induced Inflammatory Responses in Macrophages by Suppressing the Activation of the NF-κB Signaling Pathway
Source: Curr Issues Mol Biol. 2025 Oct 1;47(10):809. doi: 10.3390/cimb47100809 (PMC12562522; doi:10.3390/cimb47100809)
Supplement: Supplementary file 1 [file cimb-47-00809-s001.zip › Supplementary Figure S1.pdf]

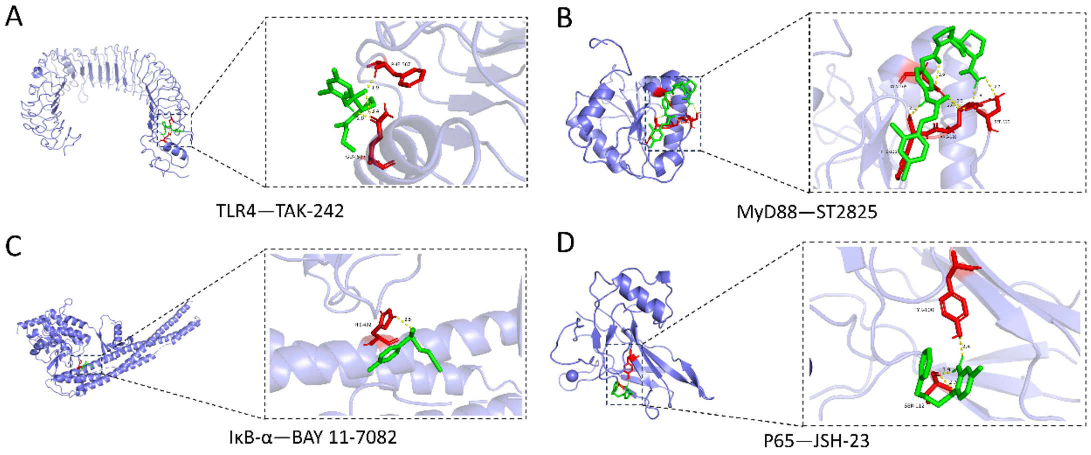

Supplementary Figure S1: Schematic diagram of molecular docking simulation between TLR4, MyD88, IκB-α, P65 -related proteins and inhibitors. (A) Molecular docking simulation diagram of the interaction between TLR4 and the inhibitor TAK-242. (B) Molecular docking simulation diagram of the interaction between MyD88 and the inhibitor ST2825. (C) Molecular docking simulation diagram of the interaction between IκB-α and the inhibitor BAY 11-7082. (D) Molecular docking simulation diagram of the interaction between P65 and the inhibitor JSH-23.
