# Supplementary material for: HCX3 Mitigates LPS-Induced Inflammatory Responses in Macrophages by Suppressing the Activation of the NF-κB Signaling Pathway
Source: Curr Issues Mol Biol. 2025 Oct 1;47(10):809. doi: 10.3390/cimb47100809 (PMC12562522; doi:10.3390/cimb47100809)
Supplement: Supplementary file 1 [file cimb-47-00809-s001.zip › Supplementary Table S1.pdf]

Supplementary Table S1: Molecular Docking Affinity and RMSD of Protein–Inhibitor Complexes.

Molecular docking	Best Affinity (kcal/mol)	Affinity Range	Minimum RMSD (l.b.)	Maximum RMSD (l.b.)
HCX3—TLR4	-7.7	-7.7~-5.7	0.000	74.272
HCX3—MyD88	-6.9	-6.9~-5.9	0.000	31.490
HCX3—IκB-α	-7.6	-7.6~-5.9	0.000	54.922
HCX3—P65	-7.6	-7.6~-6.5	0.000	30.653
TLR4—TAK-242	-5.4	-5.4~-4.6	0.000	77.101
MyD88—ST2825	-7.1	-7.1~-6.1	0.000	28.171
IκB-α—BAY 11-7082	-5.3	-5.3~-4.1	0.000	41.540
P65—JSH-23	-5.1	-5.1~-4.0	0.000	26.617

Molecular docking: The protein–inhibitor complex system involved in molecular docking. Affinity Range: The range of binding affinity values obtained from molecular docking simulations for the corresponding complex. Minimum RMSD (l.b.): The lowest root - mean - square deviation value among the docking conformations, reflecting the conformational similarity. Maximum RMSD (l.b.): The highest root - mean - square deviation value among the docking conformations, indicating the extent of conformational variation.
